# Bladder inflammatory transcriptome in response to tachykinins: Neurokinin 1 receptor-dependent genes and transcription regulatory elements

**DOI:** 10.1186/1471-2490-7-7

**Published:** 2007-05-22

**Authors:** Ricardo Saban, Cindy Simpson, Rajanikanth Vadigepalli, Sylvie Memet, Igor Dozmorov, Marcia R Saban

**Affiliations:** 1 Department of Physiology, The University Oklahoma Health Sciences Center, Oklahoma City, OK 73104, USA; 2 Daniel Baugh Institute for Functional Genomics and Computational Biology, Department of Pathology, Anatomy and Cell Biology. Thomas Jefferson University, Philadelphia PA 19107, USA; 3 Unité de Mycologie Moléculaire, URA CNRS 3012, Institut Pasteur, 75724 Paris Cedex 15, France; 4 Oklahoma Medical Research Foundation (OMRF), Arthritis and Immunology Research Program, Microarray/Euk. Genomics Core Facility, Oklahoma City, Oklahoma 73104, USA

## Abstract

**Background:**

Tachykinins (TK), such as substance P, and their neurokinin receptors which are ubiquitously expressed in the human urinary tract, represent an endogenous system regulating bladder inflammatory, immune responses, and visceral hypersensitivity. Increasing evidence correlates alterations in the TK system with urinary tract diseases such as neurogenic bladders, outflow obstruction, idiopathic detrusor instability, and interstitial cystitis. However, despite promising effects in animal models, there seems to be no published clinical study showing that NK-receptor antagonists are an effective treatment of pain in general or urinary tract disorders, such as detrusor overactivity. In order to search for therapeutic targets that could block the tachykinin system, we set forth to determine the regulatory network downstream of NK_1 _receptor activation. First, NK_1_R-dependent transcripts were determined and used to query known databases for their respective transcription regulatory elements (TREs).

**Methods:**

An expression analysis was performed using urinary bladders isolated from sensitized wild type (WT) and NK_1_R^-/- ^mice that were stimulated with saline, LPS, or antigen to provoke inflammation. Based on cDNA array results, NK_1_R-dependent genes were selected. PAINT software was used to query TRANSFAC database and to retrieve upstream TREs that were confirmed by electrophoretic mobility shift assays.

**Results:**

The regulatory network of TREs driving NK_1_R-dependent genes presented cRel in a central position driving 22% of all genes, followed by AP-1, NF-kappaB, v-Myb, CRE-BP1/c-Jun, USF, Pax-6, Efr-1, Egr-3, and AREB6. A comparison between NK_1_R-dependent and NK_1_R-independent genes revealed Nkx-2.5 as a unique discriminator. In the presence of NK_1_R, Nkx2-5 _01 was significantly correlated with 36 transcripts which included several candidates for mediating bladder development (FGF) and inflammation (PAR-3, IL-1R, IL-6, α-NGF, TSP2). In the absence of NK_1_R, the matrix Nkx2-5_02 had a predominant participation driving 8 transcripts, which includes those involved in cancer (EYA1, Trail, HSF1, and ELK-1), smooth-to-skeletal muscle trans-differentiation, and Z01, a tight-junction protein, expression. Electrophoretic mobility shift assays confirmed that, in the mouse urinary bladder, activation of NK_1_R by substance P (SP) induces both NKx-2.5 and NF-kappaB translocations.

**Conclusion:**

This is the first report describing a role for Nkx2.5 in the urinary tract. As Nkx2.5 is the unique discriminator of NK1R-modulated inflammation, it can be imagined that in the near future, new based therapies selective for controlling Nkx2.5 activity in the urinary tract may be used in the treatment in a number of bladder disorders.

## Background

Substance P belongs to the tachykinins (TKs) family of peptides involved in the peripheral and central regulation of urinary functions [1] through the stimulation of neurokinin (NK) NK_1_, NK_2_, and NK_3 _receptors [2,3]. At the urinary system level, TKs stimulate smooth muscle tone, ureteric peristalsis and bladder contractions, initiate neurogenic inflammation, and trigger local and spinal reflexes [4] aimed to maintain organ functions in emergency conditions [2]. The most studied effects produced by TKs in these systems are smooth muscle contraction [5-9], modulation of inflammation [10,11], mucus secretion, and recruitment/activation of immune cells [12]. At least in the mouse bladder, TKs are spontaneously released and their levels maintained low by the activity of neutral-endopeptidase [13]. Indeed, null deletion of NEP in mice leads to spontaneous plasma extravasation in the urinary bladder that was reversed by a recombinant of NK_1 _and bradykinin B_2 _receptors antagonists [14].

In the urinary tract, the major recognized sources of TKs are the primary afferent neurons expressing transient receptor potential vanilloid-1 receptors, which have the unique property of releasing transmitters both in the periphery (efferent function) and the spinal cord (afferent function) upon stimulation [2].

NK_1_R are the predominant subtype involved in inflammation in general [3] and may underlie persistent pain, such as that observed during chronic bladder inflammation [15]. SP activation of NK_1_R [3] induces a sequential activation of signaling pathways leading to the production of pro-inflammatory mediators [10,16,17] and pro-inflammatory cytokines such as macrophage migration inhibitory factor (MIF) that plays a major role in bladder inflammation [18].

The use of NK_1_R^-/- ^mice confirmed a central role for SP in models of bladder inflammation [19]. Indeed, NK_1_R^-/- ^mice do not mount bladder inflammatory response to antigen-complex stimulation and that NK_1_Rs are required in cystitis [19]. In this context, an up-regulation of NK_1_R was found in bladder inflammation [20] and bladder biopsies from cystitis patients present an increase in NK_1_R density [21], nerves [22], and SP-containing fibers [23]. Furthermore, the finding that sensory C fibers desensitization decreases urinary bladder hyperreflexia further supports a role for sensory peptides in this disorder [24]. In fact, NK_1_R antagonists reduce detrusor hyperreflexia caused by chemical [25] and bacterial cystitis [26], and decrease cyclophosphamide-induced inflammation [27]. In addition, changes in SP expression following cystitis may contribute to the altered visceral sensation (allodynia) and/or urinary bladder hyperreflexia in the clinical syndrome, interstitial cystitis [4].

The bulk of data obtained in experimental animal models suggests that TKs could contribute to the genesis of symptoms accompanying various diseases of the urinary tract, which includes cystitis and incontinence [28]. Indeed, a significant increase in the density of suburothelial, SP-containing nerves was found in patients with idiopathic detrusor overactivity, compared with stable controls [29,30]. Therefore, it cannot be excluded that peripheral tachykinins may be involved in pathophysiologic afferent signaling associated with detrusor overactivity [28]. However, despite promising effects in animal models, there seems to be no published clinical study showing that NK-receptor antagonists are an effective treatment of pain [31] or overactive bladder disease [28]. In addition, despite the known existence of NK_2 _receptors in the human detrusor, NK_2 _receptor antagonist does not block the non-cholinergic contraction in unstable human bladder [32].

Therefore, in order to search for putative therapeutic targets that could be manipulated to reduce the influence of the tachykinin system, we set forth to determine the regulatory network downstream of NK_1_R activation. This network is composed of genes and the transcriptional regulatory elements (TREs) that are putative binding sites for the transcription factors. In this way, we could define not only genes downstream of NK_1_R activation but also the regulators of their expression. This is based on the fact that when active transcription factors associate with TREs of their target genes, they can function to specifically repress (down-regulate) or induce (up-regulate) synthesis of the corresponding RNA. The overall hypothesis is that genes sharing the same TREs can be associated in a molecular network that would represent key pharmacological targets for modulating the influence of tachykinins in bladder diseases.

For this purpose, we used a combination of cDNA array and in silico analysis of TREs, as described previously [33]. cDNA array analysis defined the interactome of NK_1_-dependent genes by querying a web-based entry tool developed by Ingenuity Systems Inc [34]. Next, we uploaded the sequence of NK_1_-dependent genes into PAINT software and the respective TREs were identified using MATCH^® ^tool in the TRANSFAC Professional database. Genes and TREs were assembled in regulatory networks and selected TREs were confirmed by EMSA.

## Methods

### Animals

All animal experimentation described here was performed in conformity with the "Guiding Principles for Research Involving Animals and Human Beings (OUHSC Animal Care & Use Committee protocol #00-109 and #00-108). Groups of ten to twelve-week old female mice were used in these experiments. NK_1_R^-/- ^and wild type (WT, C57BL6) littermate control mice were generated by Dr. Norma P. Gerard. The colonies at OUHSC were genotyped as described previously [35].

### Antigen sensitization protocol

All mice in this study were sensitized with 1 μg DNP_4_-human serum albumin (HSA) in 1 mg alum on days 0, 7, 14, and 21, intraperitoneally (i.p.). In normal mice, this protocol induces sustained levels of IgE antibodies up to 56 days post-sensitization [36]. One week after the last sensitization, cystitis was induced. Briefly, sensitized WT and NK_1_R^-/- ^mice were anesthetized (ketamine 40 mg/kg and xylazine 2.5 mg/kg, i.p.), then transurethrally catheterized (24 Ga.; 3/4 in; Angiocath, Becton Dickson, Sandy, Utah), and the urine was drained by applying slight digital pressure to the lower abdomen. The urinary bladders were instilled with 200 μl of pyrogen-free saline or DNP_4_-OVA (1 μg/ml). One, four, and twenty-four hours after instillation, mice were sacrificed with pentobarbital (100 mg/kg, i.p.) and bladders were removed rapidly.

### Alterations at histological level

Previous results from our laboratory demonstrated a mandatory role of NK_1_R on antigen-induced cystitis [19,37]. In the present work, we also investigated whether NK_1_Rs are important for both SP- and LPS-induced cystitis. For this purpose, an additional group of NK_1_R^-/- ^and wild type (WT, C57BL6) were anesthetized as described above and challenged intravesically with 200 μl of pyrogen-free saline, SP (10 μM), or Escherichia coli LPS strain 055:B5 (Sigma, St. Louis, MO; 100 μg/ml). Twenty-four hours after instillation, mice were euthanized with pentobarbital (200 mg/kg, i.p.), and the bladders were removed rapidly for evaluation of inflammatory cell infiltrates and the presence of interstitial edema. A semi-quantitative score using defined criteria of inflammation severity was used to evaluate cystitis [37]. A cross-section of bladder wall was fixed in formalin, dehydrated in graded alcohol and xylene, embedded in paraffin, and cut serially into four 5-μm sections (8 μm apart) to be stained with hematoxylinand eosin (H&E) and Giemsa. H&E stained sections were visualized under microscope (Eclipse E600, Nikon, Lewisville, TX). All tissues were photographed at room temperature by a digital camera (DXM1200; Nikon). Exposure times were held constant when acquiring images from different groups. Images were analyzed with Image-Pro Analyzer^® ^(Media Cybernetics Inc.; Silver Spring, MD 20910). The severity of lesions in the urinary bladder was graded as follows: 1+, mild (infiltration of 0–10 neutrophils/cross-section in the lamina propria, and little or no interstitial edema); 2+, moderate(infiltration of 10–20 neutrophils/cross-section in the lamina propria, and moderate interstitial edema); 3+, severe (diffuse infiltration of >20 neutrophils/cross-section in the lamina propria and severe interstitial edema) [19,37,38]. Identification of mast cells was performed in Giemsa-stained sections [37].

### Minimum information about microarray experiments – MIAME [39]

#### a. Objective

To determine the time course of gene-expression in control and antigen-inflamed wild type and NK_1_R^-/- ^mice.

#### b. Array design

Mouse 5K Arrays (Clontech, Palo Alto, CA, Cat. #GPL151), for a complete list of genes in this array, please access Gene Expression Omnibus, GEO [40].

#### c. Animal numbers

Female WT and NK_1_R^-/- ^mice were instilled with antigen (in sensitized mice), or saline. At 1, 4, and 24 hours following stimulation, the urinary bladders were randomly distributed into the following groups: a) RNA extraction (n = 3), b) replicate of RNA extraction (n = 3), and c) morphological analysis (n = 6).

#### d. Sample preparation for cDNA expression arrays

Three bladders from each group were homogenized together in Ultraspec RNA solution (Biotecx Laboratories Inc. Houston, TX) for isolation and purification of total RNA. Mouse bladders were pooled to ensure enough RNA for gene array analysis. The justification for this approach is that there is not enough RNA in a single mouse bladder for performing cDNA array experiments, and the step of purification reduces the amount of total RNA. RNA was DNase-treated according to manufacturer's instructions (Clontech Laboratories, Palo Alto, CA), and the quality of 10 μg was evaluated by denaturing formaldehyde/agarose gel electrophoresis.

#### d. Mouse cDNA expression arrays

cDNA probes were prepared from DNase-treated RNAs obtained from each of the experiments. Five μg of DNase-treated RNA was reverse-transcribed to cDNA and labeled with [α-^32^P]dATP, according to the manufacturer's protocol (Clontech, Palo Alto, CA). The radioactively labeled complex cDNA probes were hybridized overnight to Atlas™ Mouse 5K Arrays (Clontech, Palo Alto, CA) using ExpressHyb™ hybridization solution with continuous agitation at 68°C. After two high-stringency washes, the hybridized membranes were exposed (at room temperature) to an ST Cyclone phosphor screen overnight. Spots on the arrays were quantified by BD AtlasImage™ 2.7 software (Clontech, Palo Alto, CA). The results were placed in an Excel spreadsheet.

#### f. Data normalization and analysis

Data was normalized by linear regression analysis using only genes expressed above background, as described [11,41], and the ratio of gene-expression between antigen- and saline-challenge was obtained. NK_1_R-dependent genes were selected according to the following criterion: **a**. In tissues isolated from WT mice, the expression of a particular gene should be up-regulated (ratio between antigen- and saline-treated >3.0) in at least one of the time points (1, 4, and 24 hours post challenged); **b**. in tissues isolated from NK_1_R^-/- ^mice, the expression of same gene should not be altered by antigen-challenge in any of the time points.

#### g. Database submission of microarray data

The microarray data was prepared according to "minimum information about a microarray experiment" (MIAME) recommendations [39], has been deposited in the Gene Expression Omnibus (GEO) database and can be retrieved with GEO accession number GSE2821 [42].

### Ingenuity pathways analysis

We used a novel approach [34] to fully annotate and represent NK_1_R-dependent genes by using the Ingenuity Pathways Analysis tool [43]. Using Ingenuity knowledge base network, we identified specific and canonical pathways downstream of NK_1_R activation.

### Analysis of transcriptional regulatory elements (TREs)

We employed a bioinformatics approach to hypothesize functionally relevant transcriptional regulatory elements (TREs) of NK_1_R-dependent and -independent genes. The regulatory network was determined by a combination of micro array-selected transcripts and PAINT 3.3 [44], available online [45], to query the transcription factor database (TRANSFAC) [46]. PAINT 3.3 was employed to examine 2000 base pairs of regulatory regions upstream of the transcriptional start site of each differentially expressed gene detected with the microarray. PAINT is a suite of bioinformatics and computational tools that integrate functional genomics information, as is the case of our microarray-based gene expression data, with genomic sequence and TRE data to derive hypotheses on the TREs relevant to the biological function under study.

Genbank accession numbers were used as the gene identifiers in PAINT test files. Over-representation of TREs in the matrix was calculated at levels of 0 < p <= 0.01 and 0.01 < p < 0.05 when compared to the reference (TREs regulating all genes in the original array). Employing the microarray accounts for any 'bias' present in the genes on the microarray relative to entire genome, nd guards from incorrectly concluding that certain TREs are relevant to the current experiment. The TRE hypotheses were generated from statistical enrichment analysis and were defined as those TREs that are significantly enriched such as NK_1_R-dependent and -independent genes, over random occurrence in the gene groups [44].

### Electrophoretic mobility shift assays (EMSA)

Anesthetized C57BL6 female mice were instilled with 200 μl of saline or SP (10 μM) and bladders were removed 2, 6, and 24 hours after instillation. In one additional group (zero hours), the urinary bladders were removed without instillation. Urinary bladders were placed in cold phosphate buffered saline (0°C), containing peptidase inhibitors (aprotinin, pepstatin, leupeptin at 0.01 mg/ml,) and the mucosa was dissected away from the muscle, as described previously [33]. Nuclear proteins were extracted and used for electrophoretic mobility shift assay for Nkx-2.5 and NF-kappaB.

### NF-kappaB EMSA

The NF-kappaB probe was constructed by annealing complementary synthetic oligonucleotides (5' GAT CAT GGG GAA TCC CCA 3'). Annealed probes were end-labeled with [α-^32^P] ATP (3000 Ci/mmole; GE Healthcare) and T4 polynucleotide kinase (New England Biolabs), and then purified using a G-50 column (GE Healthcare). Nuclear extracts (10 μg) were incubated with 1 ng of [^32^P] NF-kappaB double-stranded probe in 20 μl 20 mM HEPES, 70 mM KCl, 2 mM DTT, 0.01% NP-40, 4% Ficoll, 1 mg/ml BSA, and 1.4 μg poly d(I-C). For competition reactions, a 50-fold excess of unlabelled NF-kappaB probe was added to the reaction mixture, and it was incubated at room temperature for 5 minutes before the addition of the [^32^P] NF-kappaB double-stranded probe. Reaction mixtures were incubated for 20 minutes at room temperature. DNA-protein complexes were resolved on a non-denaturing 6% polyacrylamide gel at 200 V for 3 hours in 0.5 TBE (45 mM Tris-borate and 1 mM EDTA). Gels were vacuum-dried and visualized on Kodak Biomax MS Film and quantified using ImageJ Software (NIH) and statistical differences were determined using GraphPad Prism Software (GraphPad Software, San Diego, CA).

### Nkx-2.5 EMSA

Electrophoretic mobility shift assays for Nkx-2.5 were performed using a non-radioactive Gel Shift Kit (Panomics, Redwood City, CA) according to the experimental procedures provided by the manufacturer. Briefly, nuclear extracts (5 μg) were incubated with biotinylated Nkx-2.5 probe in Binding Buffer with poly d(I-C). For competition reactions, an excess of unlabelled Nkx-2.5 probe was added to the reaction mixture and it was incubated at room temperature for 5 minutes before the addition of the biotinylated Nkx-2.5 probe. Reaction mixtures were incubated for 30 minutes at 20°C. DNA-protein complexes were resolved on a non-denaturing 6% polyacrylamide gel at 200 V for 2 hours in 0.5× TBE (45 mM Tris-borate and 1 mM EDTA). The gel was then transferred onto a 0.45 μm Nytran SuperCharge membrane (Schleicher & Schuell, Keene, NH) at 300 mA for 30 minutes in 0.5× TBE. The blot was dried for 60 minutes at 80°C and then UV cross-linked for 3 minutes. After blocking, the blot was incubated with Streptavidin-HRP and developed using the substrate solutions included in the kit. Blots were visualized on Pierce CL-XPosure film (Pierce, Rockford, IL). Bands were quantified using ImageJ Software (NIH) and statistical differences were determined using GraphPad Prism Software (GraphPad Software, San Diego, CA). The sequence for the NKx-2.5 motif-containing probe, not provided by the manufacturer, is 5' AAA CAA GTC ATA ATA GGA AGC A 3'.

## Results

### Essential role of NK_1_R in cystitis

**Figure 1:**
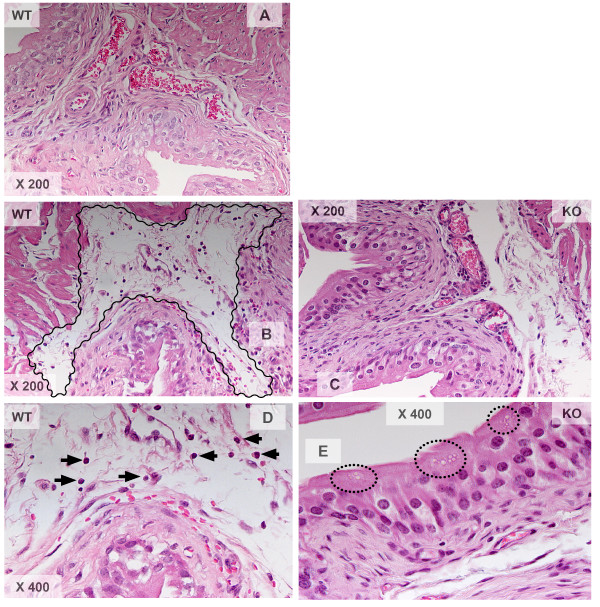
**A-E. Comparison of bladder inflammatory responses in wild type (C57BL6) and NK1-R knockout mice**. Representative photomicrographs of bladder inflammation in mice treated intravesically with 200 μl of pyrogen-free saline (1**A**), LPS (100 μg/ml; 1**B, 1C, 1D, and 1E**), or SP (10 μM; **2A, 2B, 2C, 2D, 2E, and 2F**). The urinary bladders were removed 24 hours after bladder instillation and processed for histological stains: H&E (1 **A-E and 2A-D**) and Giemsa (**2E and 2F**), (n = 6).

**Figure 2:**
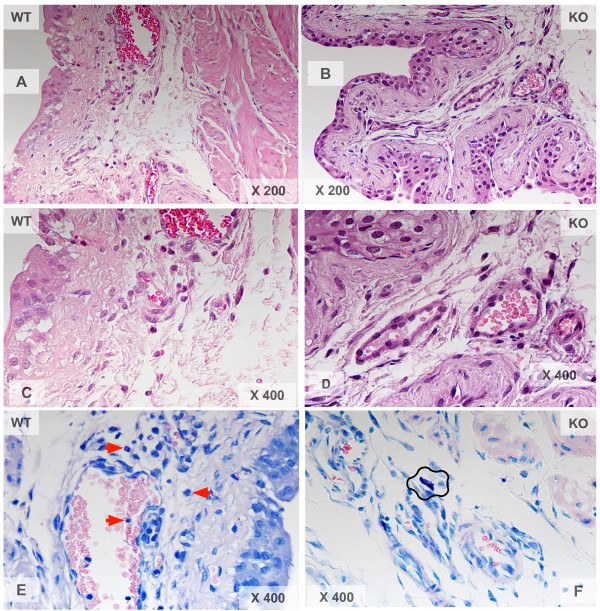
**A-F. Comparison of bladder inflammatory responses in wild type (C57BL6) and NK1-R knockout mice**. Representative photomicrographs of bladder inflammation in mice treated intravesically with 200 μl of pyrogen-free saline (1**A**), LPS (100 μg/ml; 1**B, 1C, 1D, and 1E**), or SP (10 μM; **2A, 2B, 2C, 2D, 2E, and 2F**). The urinary bladders were removed 24 hours after bladder instillation and processed for histological stains: H&E (1 **A-E and 2A-D**) and Giemsa (**2E and 2F**), (n = 6).

**Figure 3:**
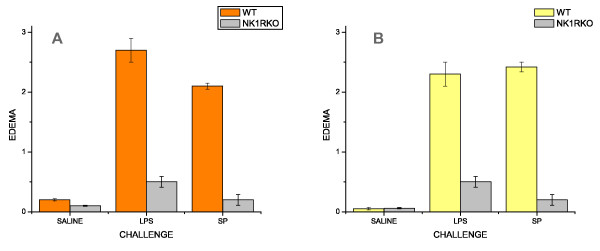
**Quantification of inflammation**. Wild type (C57BL6) and NK1-R knockout mice were treated intravesically with 200 μl of pyrogen-free saline, LPS (100 μg/ml), or SP (10 μM). The urinary bladders were removed 24 hours after bladder instillation and processed for histological stains for quantification of inflammation. **A **= Grade of edema and **B **= Grade of polymorphonuclear [PMNs] leukocytes (see material and methods).

Previous results from our laboratory demonstrated a mandatory role of NK_1_R on antigen-induced cystitis [19,37]. In the present work we also investigated whether NK_1_Rs are important for both SP- and LPS-induced cystitis. In contrast to mice treated with saline (Figure 1A), instillation of LPS into the bladder of wild type mice leads to inflammation characterized by edema (Figure 1B; black line delimits the area of sub-epithelial edema) and infiltration of inflammatory cells (Figure 1D; black arrows indicate PMNs). In contrast, the urinary bladders of NK_1_R-knockout mice failed to mount an inflammatory response to LPS (Figures 1C and 1E) despite the capacity of LPS to induce urothelial cell injury, as indicated by intra-cytoplasmatic vacuolization [47] (Figure 1E; black circles). Similar results were obtained with WT mice that mounted an inflammatory reaction to intravesicall instillation of SP as seem in H&E (Figure 2A and 2C) and Giemsa stained (Figure 2E) sections. NK_1_R-knockout failed to mount an inflammatory response to SP (Figures 2B, 2D, and 2F) with a reduced number of migrating inflammatory cells but presenting visible resident mast cells (Figure 2F; black line). Figure 3 presents the quantification of the results here described. In conclusion, these results extend our previous observation of a mandatory role for NK_1_R in antigen-induced cystitis to other pro-inflammatory mediators.

### NK_1_-dependent genes

Two hundred and nine genes fulfilled the established criteria (see material and methods) and were considered NK_1_R-dependent. In contrast, 236 genes were found to be up-regulated secondary to antigen challenge in tissues isolated from WT and NK_1_R^-/- ^mice and, therefore, were considered to be NK_1_R-independent.

### NK1-dependent transcriptional regulatory elements

**Figure 4:**
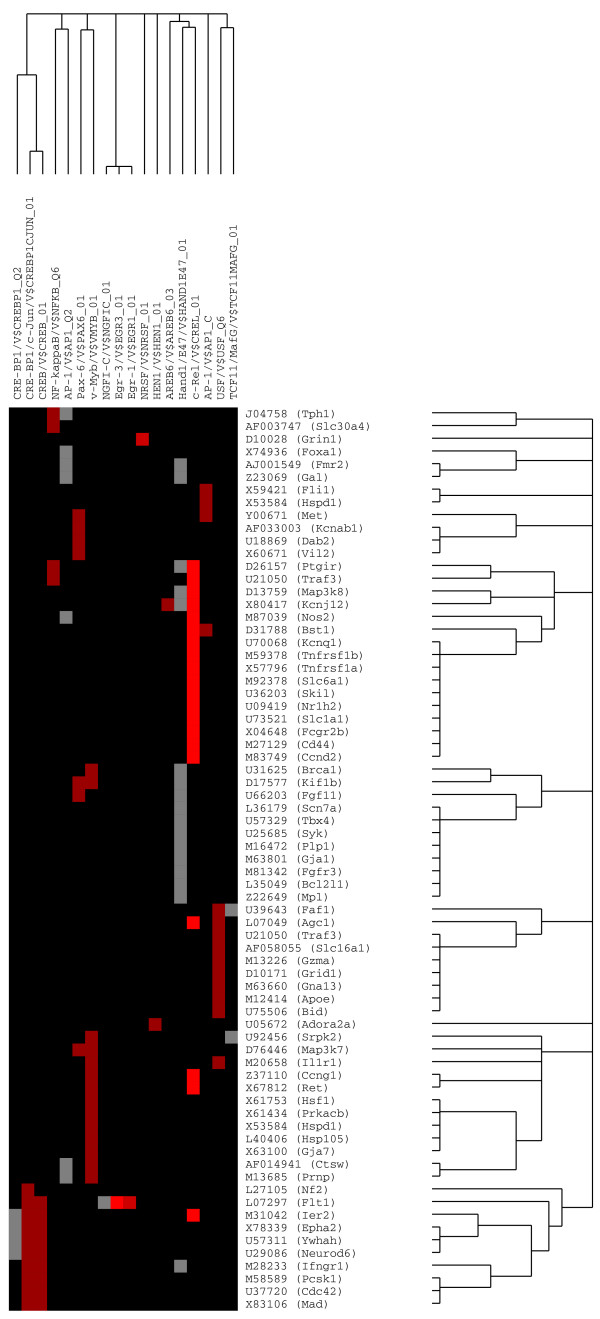
**PAINT 3.3 representation of an interaction matrix for NK_1_R-dependent genes and respective TREs**. PAINT 3.3 was employed to examine 2000 base pairs of regulatory regions upstream of the transcriptional start site of each differentially expressed gene from the microarray expression data. Genbank accession numbers were used as the gene identifiers in PAINT input files. Individual elements of the matrix are colored by the significance of the *p*-values: over-representation in the matrix is indicated in red, under-representation is indicated in cyan, and the TREs that are neither significantly over nor under-represented in the matrix are colored in gray.

**Figure 5:**
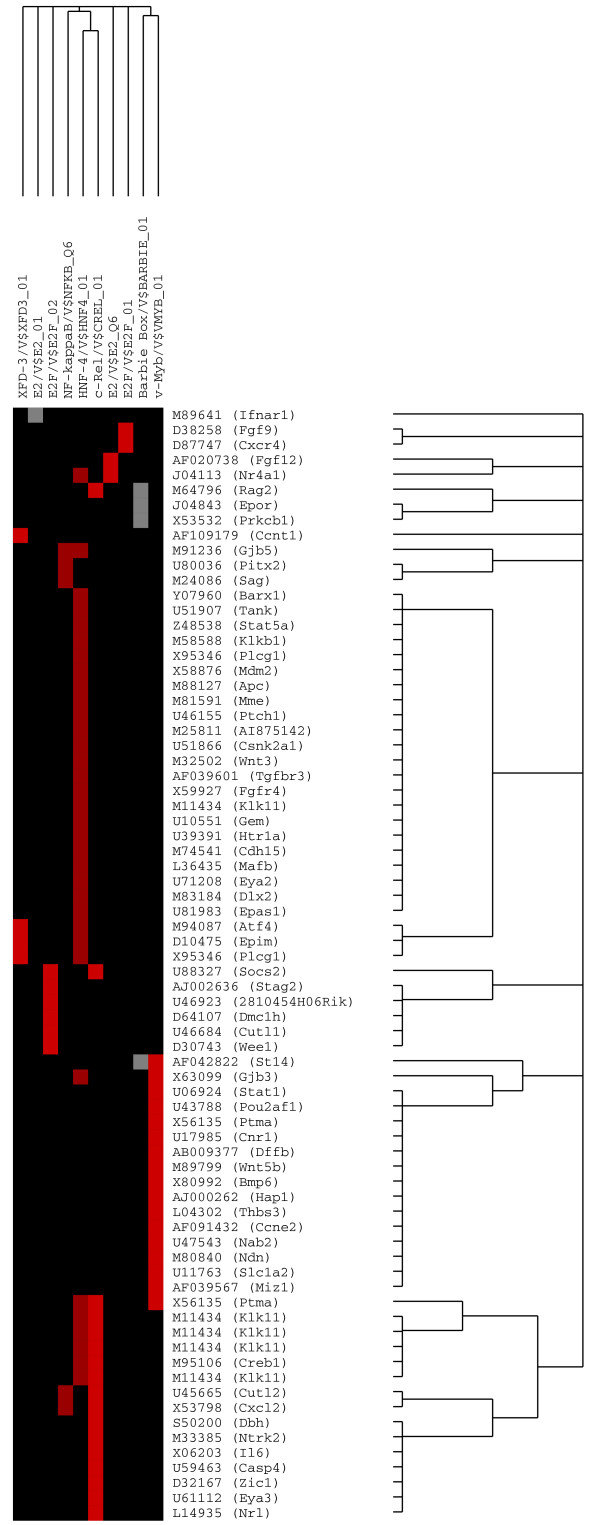
**PAINT 3.3 representation of an interaction matrix for NK_1_R-independent genes and respective TREs**. PAINT 3.3 was employed to examine 2000 base pairs of regulatory regions upstream of the transcriptional start site of each differentially expressed gene from the microarray expression data. Genbank accession numbers were used as the gene identifiers in PAINT input files. Individual elements of the matrix are colored by the significance of the *p*-values: over-representation in the matrix is indicated in red, under-representation is indicated in cyan, and the TREs that are neither significantly over nor under-represented in the matrix are colored in gray.

PAINT 3.3 was employed to examine 2000 base pairs of regulatory regions upstream of the transcriptional start site of each differentially expressed gene from the microarray expression data. Genbank accession numbers were used as gene identifiers in PAINT input files. For the NK_1_R-dependent genes, out of a total of 209 genes from the expression analysis, only 153 genes had corresponding upstream sequence information owing to the incomplete nature of genomic annotation. A total of 87 TREs were identified on these sequences using MATCH^® ^tool in the TRANSFAC Professional database. Similarly, for the NK_1_R-independent genes, only 188 promoters were retrieved for the 236 genes from expression analysis. A total of 88 TREs were identified on these sequences using MATCH^®^. These TREs were examined in the enrichment analysis to derive regulatory network hypotheses. The resulting candidate interaction network can be visualized as an interaction matrix where the individual elements of the matrix are color-coded based on the *p*-values for statistical enrichment. A *p*-value threshold of 0.07 was used to filter the enrichment analysis results to derive the regulatory network hypotheses. These results are shown in Figure 4 for NK_1_R-dependent genes and in Figure 5 for NK_1_R-independent genes.

### Regulatory network downstream of NK_1_R activation

**Figure 6:**
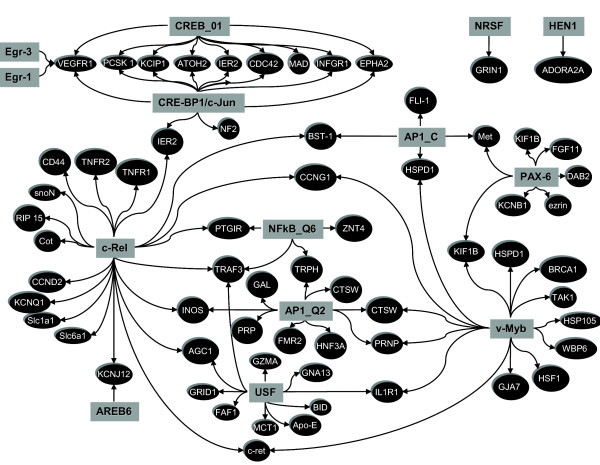
**Regulatory Network downstream NK_1 _receptor activation**. Hypothesized regulatory network corresponding to the NK_1_R-dependent genes and respective TREs. A complete list of genes and TREs is summarized on Table 1 (Additional file 1).

Figure 6 depicts the hypothesized regulatory network corresponding to the NK_1_R-dependent genes whose TRE were found to be over-represented (0.01 < p < 0.05) in this set when compared to a reference (all genes in the array). cRel was the predominant TRE, driving 22% of the NK_1_R-dependent genes. The order of predominance for the different TREs was: cRel, v-Myb, CRE-BP1/c-Jun, USF, AP-1_Q2, Pax-6, AP-1_C, NF-kappaB_Q6, Efr-1, Egr-3, and AREB6 (Figure 6 and Table 1 [additional file 1]).

### Ingenuity pathways analysis

**Figure 7:**
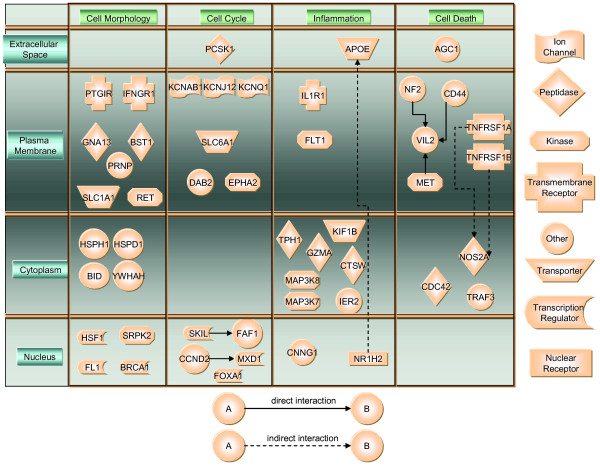
Ingenuity Pathways annotation of NK_1_R-dependent genes.

In order to make sense of the vast information generated by cDNA array expression, we used the recently developed Ingenuity Pathways Knowledge Base [34] to design pathways of NK1R-dependent genes and to query canonic pathways regarding the relative importance of each of the transcripts (Figure 7). As a result, genes were localized to different compartments depending on the predominant expression of the protein they encoded (Figure 7). In this way, four compartments are depicted: extracellular space, plasma membrane, cytoplasm, and nucleus. According to their primary function, ingenuity grouped NK_1_-dependent genes into 4 different networks: cell morphology, cell cycle, inflammation, and cell death. However, a strong degree of overlapping between cell morphology, cell cycle, cell death, and cancer was observed. In contrast, genes involved in inflammation were also listed to be involved in embryonic development. The NK1R-dependent genes were significantly correlated with the following canonical signaling pathways: p38 MAPK, NF-kappaB, PPAR, IL-6, death receptor, apoptosis, and SAPK/JNK (Table 2, [additional file 2]).

Overall NK_1_R-dependent genes were classified following the biological processes with which they are involved (GO anthology): apoptosis (GZMA, TNFRSF1B, TNFRSF1A, TRAF3, NOS2A, and BID); cell adhesion/hyaluronic acid binding (CD44 and AGC1); cell cycle (CCND2 and CCNG1); cell-cell signaling (FGF11 and GJA7); cytokinesis (CDC42 and KIF1B); development (FMR2); extracellular transporters & carriers (APOE); G-protein coupled receptors (GNA13 and PTGIR); growth factors (MXD1); heat shock proteins (PRNP, HSPH1, and HSPD1); immune response (BST-1, CTSW, and IL1R1); interferons (INFGR1) intracellular kinases (WBP6); intracellular transducers (MAP3K7); kinase activators & inhibitors (YWHAH); membrane channels (KCNAB1, KCNJ12, KCNQ1, and SLC30A4); nucleotide metabolism (PCSK1); oncogenes & tumor suppressors (BRCA1, MAP3K8, RET, VIL2, FLI1, MET, NF2, and VEGFR1); receptor mediated endocytosis (DAB2); receptor tyrosine kinase (EPHA2); regulation of transcription (NEUROD6); serotonin biosynthesis (YTPH1); symporters & antiporters (SLC16A1 and SLC1A1); transcription activators & repressors (FOXA1, HSF1, IER2, and NR1H2); and synaptic transmission (GRID1).

### Comparing NK1R-dependent and -independent genes

**Figure 8:**
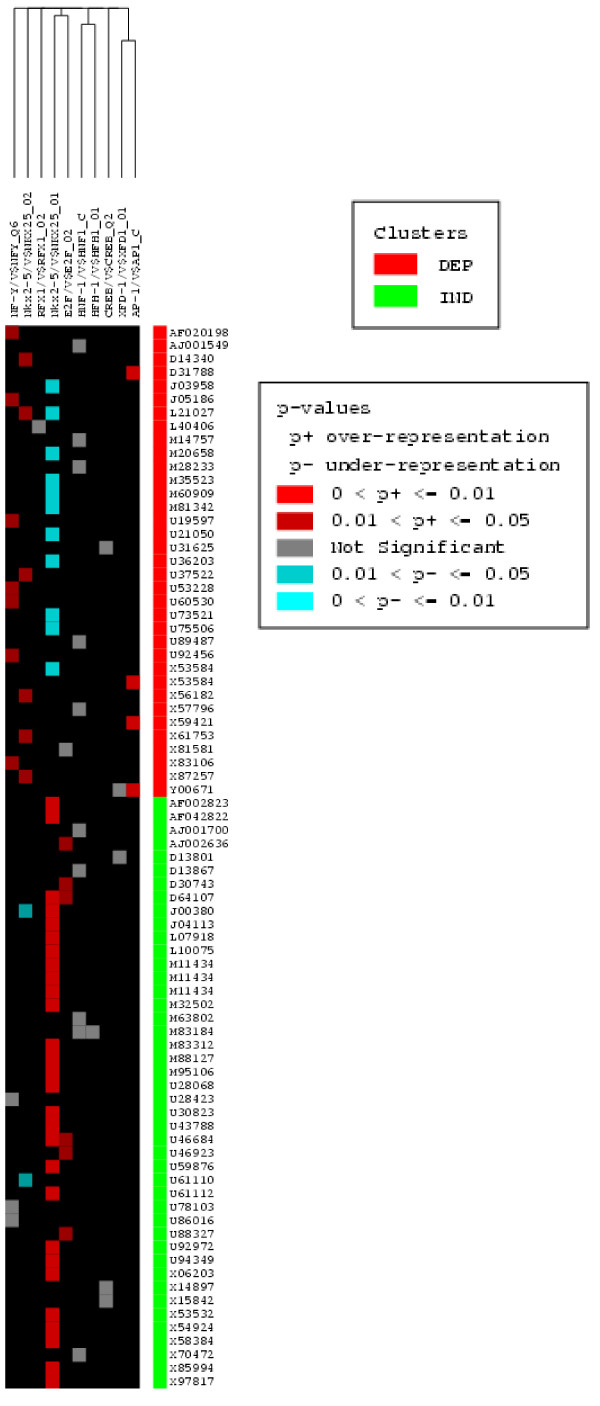
**Direct comparison of TREs driving NK_1_R-dependent and-independent genes**. The whole set of genes that were up-regulated at least 3-fold during bladder inflammation were analyzed in PAINT 3.3. These genes were divided into two groups: NK_1_R-dependent and NK_1_R-independent. A p-value threshold of 0.05 was used to derive statistically enriched regulatory elements in these two gene groups. Note that AP-1 is over represented in the set of NK1R-dependent genes, whereas it is not significantly correlated with NK_1_R-independent transcripts. Also note that Nkx-2.5 matrix _01 was found over represented in the set of NK_1_R-independent transcripts (colored in red) and under-represented or suppressed in the set of NK_1_R-dependent TRs (colored in cyan). In contrast, Nkx-2.5 matrix _02 had the completely opposite behavior.

The whole set of genes that were up-regulated at least 3-fold during inflammation including NK_1_R-dependent and NK_1_R-independent genes were analyzed in PAINT. A *p*-value threshold of 0.05 was used to derive statistically enriched regulatory elements in these two gene groups (Figure 8). The enrichment in this case was obtained by using the interaction matrix for the combined gene list as a reference. This enabled us to contrast the two gene groups relative to each other and characterize those regulatory elements that are specific to one group or the other. We observed differential enrichment of several regulatory elements including over-expression of AP1 specific in the NK_1_R-dependent genes, as we previously suggested [41] (Figure 8). However, the most striking finding of this analysis was the revelation that Nkx-2.5, a murine homeo box gene, is a unique discriminator of NK_1_R-dependent genes. Nkx-2.5 matrix _01 was found over represented in the set of NK_1_R-independent transcripts (colored in red) and under-represented or suppressed in the set of NK1R-dependent TREs (colored in cyan). In contrast, Nkx-2.5 matrix _02 had the completely opposite behavior since it was over-represented in NK1R-dependent and under-represented in the NK_1_R-independent set. Table 3 [additional file 3] lists all genes under the control of both Nkx-2.5 matrix _01 and matrix _02. With the exception of A10 (L21027) and EGF (J00380) that were associated with both matrixes, a unique set of genes is correlated with Nkx-2.5 matrix _01 and Nkx-2.5 matrix _02.

### Intravesical instillation of SP stimulates NF-kappaB and Nkx-2.5 translocation in the bladder mucosa

**Figure 9:**
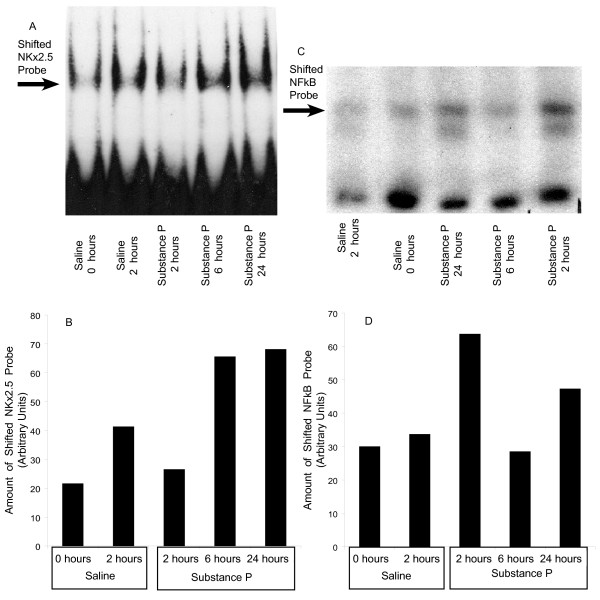
**Intravesical SP stimulates NKx-2.5 and NF-kappaB activity**. Anesthetized mice were instilled with 200 μl of saline or SP (10 μM) and bladders were removed 2, 6, and 24 hours after instillation. In one additional group (zero hours) the urinary bladders were removed without instillation. Urinary bladders were placed in cold phosphate buffered saline (0°C) containing protease inhibitors, and the mucosa was dissected away from the muscle. Nuclear proteins were extracted and used for electrophoretic mobility shift assay for NKx-2.5 (**A**) and NF-kappaB (**C**). Amount of shifted Nkx-2.5 (**B**) and NF-kappaB (**D**) probes were quantified as described in materials and methods.

To determine whether activation of NK_1_R increases the transcriptional activities of NF-kappaB and Nkx-2.5, an EMSA was performed. Results show that instillation of SP into the mouse bladder increased the amount of shifted Nkx-2.5 and NF-kappaB probes that peaked at 24 hours post stimulation (Figure 9 A-D). Because of the high level of expression of both NKx-2.5 and NF-kappaB in the bladders pre-treated with saline, in an additional group, urinary bladders were removed without insertion of the catheter or fluid instillation. Interestingly, in this group (0 hours) almost the same degree of constitutive transcriptional activities was observed.

## Discussion

It has to be taken into consideration that the approach of this study permitted the generation of new testable hypothesis rather than the more traditional hypothesis-driven research. Indeed, the major question being answered by this work is what TREs can be therapeutically targeted for reducing the influence of tachykinins in bladder disorders? The method introduced here, supplements the standard procedure of multiple paired comparisons used in microarray analysis by associating the expression level of each gene in the experimental group with a family transcription regulatory elements[33] and to compare with the occurrence of each TRE in a reference file (all genes in the array). This bottom-up approach builds mechanistic models for each individual case, e.g., identifying the binding sites for selected genes and their respective TREs [48], then specifies the role of each TRE in the network generating a testable hypothesis for the network downstream of NK_1_R activation. Next, we used EMSA to confirm that selected TREs (NF-kappaB and NKx-2.5) are indeed part of the molecular network downstream of NK_1_R activation.

An extended family of TREs was significantly correlated with NK_1_R-dependent genes. Those included c-Rel, NF-kappaB_Q6, PAX-6, CREB_01, CRE-BP1/c-Jun, and v-Myb (Figure 4). However, most of the studies on transcription regulatory elements in urology are related to oncology, which makes it difficult to further illustrate the clinical relevance of our findings. Therefore, we are discussing only the most relevant TREs that modulate bladder inflammatory responses to SP.

AP1 was among the NK1R-dependent TREs. We have provided evidence for a predominant role for AP1 controlling highly expressed NK_1_R-dependent genes [41]. In the present work, we confirmed the regulatory relationships between AP1 (AP1_Q2 and AP1_C) and NK_1_R-dependent genes. It is known that the activation of MAPK (JNK, p38) and NF-kappaB signaling pathways leads to the activation of AP-1 and, consequently, the inflammation [49]. The present results extend these findings to the urinary bladder where these pathways can be explored as potential therapeutic targets to decrease the symptoms of cystitis.

NF-kappaB is believed to trigger both the onset and the resolution of inflammation. NF-kappaB activity is correlated with bladder cancer [50-52] and bladder urothelial cells respond to insults with a translocation of NF-kappaB [53] leading ultimately to an increased NK_1_R expression [54]. Our present work confirms previous indication that Tachykinins, such as SP, activate NF-kappaB translocation [55,56]. Indeed, in the urinary bladder, activation of NK_1_R by SP induces NF-kappaB translocation, as seen by EMSA results (Figure 9), and up-regulation of pro-inflammatory genes, such as the encoding prostaglandin I_2 _receptor (Figure 6).

Another TRE over-expressed in the NK_1_R-dependent cluster was the upstream stimulatory factor (USF). Although widely expressed, USF can mediate tissue-specific transcripts. USF is stimulated by glucose in murine mesangial cells, binds to TGF-β1 promoter, contributes to TGF-β1 expression, and may play a role in diabetes-related gene regulation in the kidney [57].

However, the most impressive switch between NK_1_R-dependent and independent transcripts was the one observed with two different matrixes of Nkx-2.5 (_01 and _02). Both Nkx-2.5_01 and _02 are binding sites derived from mouse sequences [58,59]. Nkx-2.5 is a murine homeobox named tinman homeodomain factor and is considered to be a new member of the sub-family of homeobox genes related to the Drosophila [58]. Nkx-2.5 is proposed as a valuable marker in the analysis of mesoderm development [59]. It was first described as an essential transcription factor for normal heart morphogenesis, myogenesis, and function [60]. However, more recently it was shown that Nkx-2.5 is required for the expression of atrial natriuretic peptide [61] and, along with NF-kappaB, is part of the brain natriuretic peptide promoter [62]. Outside of the heart, this element is important in vessel remodeling [63], skeletal myogenesis [64], and pyloric sphincter development [65]. Other sites of Nkx-2.5 expression include pharyngeal endoderm and its derivatives, branchial arch epithelium, stomach, spleen, pancreas and liver [66].

To our knowledge, this is the first report describing a role for Nkx-2.5 in the urinary tract. In the presence of NK_1_R, Nkx-2.5 _01 was significantly correlated with 36 transcripts which included several candidates for mediating bladder development (FGF) and inflammation (PAR-3, IL-1R, IL-6, NGF, TSP2) (Table 3, [additional file 3]). In the absence of NK_1_R, the matrix _02 had a predominant participation driving 8 transcripts, which includes those involved in cancer (EYA1, Trail, HSF, and ELK-1), smooth-to-skeletal muscle trans-differentiation, and Z01, a tight-junction protein, expression (Table 3, [additional file 3]).

An interesting finding was the constitutive translocation of NKx-2.5 and NF-kappaB in the bladder mucosa. One possible explanation was that mechanical stimulation caused by instillation of saline caused the shift. Therefore, an additional control group was added in which the bladder was removed without instillation. This group (0 hours) also presented a certain amount of shifted NKx-2.5 and NF-kappaB probes. An alternative explanation for these results is that mechanical isolation of the bladder mucosa caused the translocation of both transcription factors. We, therefore, generated preliminary results using an urothelial cell line (J82) which indicated a constitutive activation of both NF-kappaB and NKx-2.5 in the absence of overt stimulation (data not shown). Therefore, we suggest that the bladder mucosa/urothelium might present a constitutive activation of both transcription factors. The similarity of basal translocation of NKx-2.5 and NF-kappaB translocation in the urinary bladder may be related to an overlap of binding motifs in some genes. Indeed, others have shown an overlap of conserved DNA binding motifs including AP-1 sites, NF-kappaB, GATA, and Nkx-2.5 in promoter regions of genes, such as MMP13 [67].

## Conclusion

This work indicates an overriding participation of NK_1_R in bladder inflammation, provides a working model for the involvement of transcription regulators such as NF-kappaB, and Nkx-2.5, and evokes testable hypotheses regarding a role for tachykinins in the urinary tract pathology. It remains to be determined whether the control of Nkx-2.5 activity by gene silencing or double mutant negative blockers will ameliorate the clinical manifestations of cystitis.

## Abbreviations

AP-1 = activator protein-1; ELK-1 = Elk-1 ets-related proto-oncogene; EMSA = electrophoretic mobility shift assay; EYA1 = eyes absent homolog 1; FGF = fibroblast growth factor; HSF1 = transcription factor 1 for heat shock gene; IL-1R = interleukin-1 receptor; IL-6 = Interleukin-6; LPS = lipopolysaccharide; PAR-3 = proteinase-activated receptor 3; NGF = nerve growth factor; Trail = TNF-related apoptosis inducing ligand; TSP2 = thrombospondin 2; and SP = substance P.

## Competing interests

The author(s) declare that they have no competing interests.

## Authors' contributions

**RS **conceived of the study and drafted the manuscript. **CS **performed the EMSAs. **RV **consulted **RS **regarding PAINT and TRANSFAC analysis. **SM **introduced EMSA experiments in RS's laboratory and helped **CS **with the interpretation on EMSA results. **ID **normalized and analyzed the gene-array results. **MRS **participated in the experimental design, carried out the animal experiments, extracted the RNA and performed gene array results.

## Pre-publication history

The pre-publication history for this paper can be accessed here:



## Supplementary Material

Additional File 1 Table 1 – Annotation of NK1R-Dependent Genes and Respective Transcription Regulators. Click here for file

Additional File 2 Table 2 – NK1R-dependent genes involved in Canonic Pathways Click here for file

Additional File 3 Table 3 – Genes regulated by Nkx2-5/V$NKX25_01 and Nkx2-5/V$NKX25_02 Click here for file
