# Supplementary material for: Bladder inflammatory transcriptome in response to tachykinins: Neurokinin 1 receptor-dependent genes and transcription regulatory elements
Source: BMC Urol. 2007 May 22;7:7. doi: 10.1186/1471-2490-7-7 (PMC1888709; doi:10.1186/1471-2490-7-7)
Supplement: Additional File 1 — Table 1 – Annotation of NK1R-Dependent Genes and Respective Transcription Regulators. [file 1471-2490-7-7-S1.pdf]

TABLE 1. NK1R-Dependent Genes and Respective Transcription Regulators

Abbrev	GenBank ID	Gene Name	GO Biological Process	Family	LOCATION	TER (probability)			
AGC1	L07049	aggrecan 1	Cell Adhesion/hyaluronic acid binding	Other	Extracellular Space	c-Rel (0.0004)	USF (0.06)		
APOE	M12414	apolipoprotein E	Extracellular Transporters & Carriers	Transporter	Extracellular Space	USF (0.06)			
BID	U75506	BH3 interacting domain death agonist	Apoptosis	Other	Cytoplasm	USF (0.06)			
BRCA1	U31625	breast/ovarian cancer susceptibility locus 1	Oncogenes & Tumor Suppressors	Transcription regulator	Nucleus	v-Myb (0.05)			
BST1	D31788	bone marrow stromal cell antigen 1	Immune response	Enzyme	Plasma Membrane	c-Rel (0.0004)	AP-1_C (0.05)		
CCND2	M83749	cyclin D2	Cell cycle	Other	Nucleus	c-Rel (0.0004)			
CCNG1	Z37110	cyclin G1	Cell cycle	Other	Nucleus	c-Rel (0.0004)	v-Myb (0.05)		
CD44	M27129	CD44 antigen	Cell Adhesion/hyaluronic acid binding	Other	Plasma Membrane	c-Rel (0.0004)			
CDC42	U37720	cell division cycle 42	cytokinesis	Enzyme	Cytoplasm	CREB_01 (0.06)	CRE-BP1 (0.07)		
CTSW	AF014941	cathepsin W	Immune response	Peptidase	Cytoplasm	v-Myb (0.05)	AP-1_Q2 (0.05)		
DAB2	U18869	disabled homolog 2	Receptor mediated endocytosis	Other	Plasma Membrane	Pax-6 (0.05)			
EPHA2	X78339	ephrin type A receptor 2	Receptor tyrosine kinase	Kinase	Plasma Membrane	CREB_01 (0.06)	CRE-BP1 (0.07)		
FAF1	U39643	Fas (TNFRSF6) associated factor 1	Apoptosis	Other	Nucleus	USF (0.06)			
FGF11	U66203	fibroblast gowth factor 11	Cell-cell signaling	Signal Transduction		Pax-6 (0.05)			
FLI1	X59421	ets-related proto-oncogene	Oncogenes & Tumor Suppressors	Transcription regulator	Nucleus	AP-1_C (0.05)			
VEGFR1	L07297	vascular endothelial growth factor receptor 1	Oncogenes & Tumor Suppressors	Kinase	Plasma Membrane	CREB_01 (0.06)	CRE-BP1 (0.07)	Egr-3 (0.006)	Egr-1 (0.01)
FMR2	AJ001549	fragile X mental retardation syndrome 2	Development			AP-1_Q2 (0.05)			
FOXA1	X74936	hepatocyte nuclear factor 3 alpha	Transcription Activators & Repressors	Transcription regulator	Nucleus	AP-1_Q2 (0.05)			
GJA7	X63100	gap junction alpha 7 protein	Cell-cell signaling	Transporter	Plasma Membrane	v-Myb (0.05)			
GNA13	M63660	guanine nucleotide binding protein	GPCR	Enzyme	Plasma Membrane	USF (0.06)			
GRID1	D10171	glutamate receptor, ionotropic, delta 1	Synaptic transmission			USF (0.06)			
GZMA	M13226	granzyme A (EC 3.4.21.78)	Apoptosis	Peptidase	Cytoplasm	USF (0.06)			
HSF1	X61753	heat shock transcription factor 1	Transcription Activators & Repressors	Transcription regulator	Nucleus	v-Myb (0.05)			
HSPD1	X53584	heat shock 60kDa protein 1	Heat Shock Proteins	Other	Cytoplasm	v-Myb (0.05)	AP-1_C (0.05)		
HSPH1	L40406	heat shock protein 105	Heat Shock Proteins	Other	Cytoplasm	v-Myb (0.05)			
IER2	M31042	T-lymphocyte activated protein	Transcription Activators & Repressors	Other	Cytoplasm	CREB_01 (0.06)	CRE-BP1 (0.07)	c-Rel (0.0004)	
IFNGR1	M28233	interferon-gamma receptor	Interferons	Transmembrane Receptors	Plasma Membrane	CREB_01 (0.06)	CRE-BP1 (0.07)		
IL1R1	M20658	interleukin-1 receptor	Immune response	Transmembrane Receptors	Plasma Membrane	v-Myb (0.05)	USF (0.06)		
KCNAB1	AF033003	K+ channel beta-1 subunit shaker-related	Membrane Channels	Ion Channel	Plasma Membrane	Pax-6 (0.05)			
KCNQ1	U70068	K+ KQT-like 1	Membrane Channels	Ion Channel	Plasma Membrane	c-Rel (0.0004)			
KIF1B	D17577	kinesin heavy chain member 1B	cytokinesis	Transporter	Cytoplasm	Pax-6 (0.05)	v-Myb (0.05)		
KNCJ12	X80417	K+ channel, subfamily J, member 12	Membrane Channels	Ion Channel	Plasma Membrane	c-Rel (0.0004)	AREB6 (0.05)		
MAP3K7	D76446	mitogen-activated protein kinase kinase 7/TGFb-activated kinase 1	Intracellular Transducers	Enzyme	Cytoplasm	v-Myb (0.05)			
MAP3K8	D13759	Cot proto-oncogene	Oncogenes & Tumor Suppressors	Kinase	Cytoplasm	c-Rel (0.0004)			
MET	Y00671	c-Met proto-oncogene	Oncogenes & Tumor Suppressors	Kinase	Plasma Membrane	Pax-6 (0.05)	AP-1_C (0.05)		
MXD1	X83106	MAX dimerization protein	Growth Factors	Transcription regulator	Nucleus	CREB_01 (0.06)	CRE-BP1 (0.07)		
NEUROD6	U29086	neurogenic differentiation 6 (neuronal helix-loop-helix)	Regulation of transcription	Transcription regulator	Nucleus	CREB_01 (0.06)	CRE-BP1 (0.07)		
NF2	L27105	neurofibromatosis type 2 susceptibility	Oncogenes & Tumor Suppressors	Other	Plasma Membrane	CRE-BP1 (0.07)			
NOS2A	M87039	nitric oxide synthase 2A	Apoptosis	Enzyme	Cytoplasm	c-Rel (0.0004)	AP-1_Q2 (0.05)		
NR1H2	U09419	nuclear receptor subfamily 1/ LRX	Transcription Activators & Repressors	Ligand-dependent nuclear receptor	Nucleus	c-Rel (0.0004)			
PCSK1	M58589	proprotein convertase subtilisin/kexin	Nucleotide Metabolism	Peptidase	Extracellular Space	CREB_01 (0.06)	CRE-BP1 (0.07)		
PRNP	M13685	major prion protein precursor	Heat Shock Proteins	Other	Plasma Membrane	v-Myb (0.05)	AP-1_Q2 (0.05)		
PTGIR	D26157	prostaglandin I2 receptor	GPCR	GPCR	Plasma Membrane	c-Rel (0.0004)	NF-kB (0.05)		
RET	X67812	c-ret proto-oncogene	Oncogenes & Tumor Suppressors	Kinase	Plasma Membrane	c-Rel (0.0004)	v-Myb (0.05)		
SKIL	U36203	ski-like oncogene	Oncogenes & Tumor Suppressors	Transcription regulator	Nucleus	c-Rel (0.0004)			
SLC16A1	AF058055	Monocarboxylate transporter	Symporters & Antiporters	Transporter	Plasma Membrane	USF (0.06)			
SLC1A1	U73521	high affinity glutamate transporter	Symporters & Antiporters	Kinase	Nucleus	c-Rel (0.0004)			
SLC30A4	AF003747	Soluble carrier family 30 (zinc family) member 4	Membrane Channels			NF-kB (0.05)			
SLC6A1	M92378	soluble carrier 6/GABA transporter 1	Symporters & Antiporters	Transporter	Plasma Membrane	c-Rel (0.0004)			
SRPK2	U92456	SFRS protein kinase 2/WBP6	Intracellular Kinases	Kinase	Nucleus	v-Myb (0.05)			
TNFRSF1A	X57796	tumor necrosis factor receptor 1	Apoptosis			c-Rel (0.0004)			
TNFRSF1B	M59378	tumor necrosis factor receptor 2	Apoptosis			c-Rel (0.0004)			
TRAF3	U21050	TNF receptor-associated factor 3	Apoptosis	Other	Cytoplasm	c-Rel (0.0004)	USF (0.06)	NF-kB (0.05)	
VIL2	X60671	ezrin, villin 2	Oncogenes & Tumor Suppressors	Other	Plasma Membrane	Pax-6 (0.05)			
YTPH1	J04758	tryptophan hydroxylase	Serotonin biosynthesis	Enzyme	Unkown	NF-kB (0.05)	AP-1_Q2 (0.05)		
YWHAH	U57311	tyrosine 3-mooxygenase/ eta polypeptide	Kinase Activators & Inhibitors	Other	Cytoplasm	CREB_01 (0.06)	CRE-BP1 (0.07)		
