# Supplementary material for: Bladder inflammatory transcriptome in response to tachykinins: Neurokinin 1 receptor-dependent genes and transcription regulatory elements
Source: BMC Urol. 2007 May 22;7:7. doi: 10.1186/1471-2490-7-7 (PMC1888709; doi:10.1186/1471-2490-7-7)
Supplement: Additional File 2 — Table 2 – NK1R-dependent genes involved in Canonic Pathways [file 1471-2490-7-7-S2.pdf]

Table 2. NK1R-dependent genes involved in Canonic Pathways.

		Pathways						
**Abbrev**	**Gene Name**	**NFKB**	**p38 MAPK**	**PPAR**	**IL-6**	**Death Receptor**	**Apoptosis**	**SAPK/JNK**
BID	BH3 interacting domain death agonist					Yes	Yes	
CDC42	cell division cycle 42							Yes
GNA13	guanine nucleotide binding protein							Yes
IL1R1	interleukin-1 receptor	Yes	Yes	Yes	Yes			
MAP3K7	mitogen-activated protein kinase kinase 7/TGFb-activated kinase 1	Yes	Yes	Yes	Yes			Yes
MAP3K8	Cot proto-oncogene	Yes						
TNFRSF1A	TNF receptor 1	Yes	Yes	Yes	Yes	Yes	Yes	
TNFRSF1B	TNF receptor 2		Yes	Yes	Yes	Yes	Yes	
TRAF3	TNF receptor-associated factor 3	Yes						
