# Supplementary material for: Bladder inflammatory transcriptome in response to tachykinins: Neurokinin 1 receptor-dependent genes and transcription regulatory elements
Source: BMC Urol. 2007 May 22;7:7. doi: 10.1186/1471-2490-7-7 (PMC1888709; doi:10.1186/1471-2490-7-7)
Supplement: Additional File 3 — Table 3 – Genes regulated by Nkx2-5/V$NKX25_01 and Nkx2-5/V$NKX25_02 [file 1471-2490-7-7-S3.pdf]

Table 3. Genes regulated by Nkx2-5/V$NKX25_01 and Nkx2-5/V$NKX25_02

Nkx2-5/V$NKX25_01		
**Abbrev**	**GenBank ID**	**Gene Name**
**A10**	**L21027**	transcription factor A10
alpha-NGF	M11434	7S nerve growth factor alpha
APC	M88127	adenomatous polyposis coli protein
BID	U75506	BH3 interacting domain death agonist
Bub1	AF002823	Bub1 mitotic checkpoint kinase
CD 40L	M83312	CD 40L receptor
CRABP2	M35523	cellular retinoic acid-binding protein II
CREB1	M95106	cAMP responsive element binding protein 1
CUTL1	U46684	CUT-related homeobox CUX-1
DCM1	D64107	meiotic recombination protein
EAAC1	U73521	Excitatory amino acid transporter 3
**EGF**	**J00380**	epidermal growth factor
EPITHIN	AF042822	Epithin
EYA3	U61112	eyes absent homolog 3
FGF3	M81342	fibroblast growth factor 3 precursor
Gsta2	J03958	glutathione S-transferase A
HMR	J04113	nuclear hormone receptor
HSP65	X53584	heat shock 60-kDa protein
IL-1 R	M20658	interleukin-1 receptor
IL-6	X06203	Interleukin-6 precursor
MEF2A	U30823	myocyte-specific enhancer factor 2A
MFNG	U94349	manic fringe homolog precursor
Mgcm1	U59876	glial cells missing gene homolog
NEUROD1	U28068	neurogenic differentiation factor 1
NF1	X54924	neurofibromatosis 1
PAR3	U92972	proteinase-activated receptor 3
PKCB	X53532	protein kinase C beta II
POU	U43788	POU domain (class 2) associated factor 1
RARA	M60909	retinoic acid receptor alpha
SEMA3C	X85994	semaphorin IIIC
SEMAF	X97817	semaphorin F
SMBP2	L10075	DNA-binding protein
snoN	U36203	ski-related oncogene
THAM	X58384	thymocyte-activating molecule
TRAF3	U21050	TNF receptor-associated factor 3
TSP2	L07918	thrombospondin 2 precursor
WNT3	M32502	wingless-related MMTV integration site 3

Nkx2-5/V$NKX25_02		
**Abbrev**	**GenBank ID**	**Gene Name**
**A10**	**L21027**	transcription factor A10
**EGF**	**J00380**	epidermal growth factor
ELK-1 PO	X87257	Elk-1 ets-related proto-oncogene
EYA1	U61110	eyes absent homolog 1
Hsf1	X61753	transcription factor 1 for heat shock gene
Myf5	X56182	myogenic factor 5
TRAIL	U37522	TNF-related apoptosis inducing ligand
ZO1	D14340	tight junction protein

Genes in bold face are regulated by both _01 and _02 matrixes
